# Computer Programming E-Learners’ Personality Traits, Self-Reported Cognitive Abilities, and Learning Motivating Factors

**DOI:** 10.3390/brainsci11091205

**Published:** 2021-09-13

**Authors:** Aiste Dirzyte, Aivaras Vijaikis, Aidas Perminas, Romualda Rimasiute-Knabikiene, Lukas Kaminskis, Giedrius Zebrauskas

**Affiliations:** 1 Faculty of Creative Industries, Vilnius Gediminas Technical University, Saulėtekio Ave. 11, 10221 Vilnius, Lithuania; 2 Institute of Psychology, Mykolas Romeris University, Ateities 20, 08303 Vilnius, Lithuania; aivarasvijaikis@gmail.com (A.V.); romualda.rimasiute@gmail.com (R.R.-K.); 3 Department of Psychology, Vytautas Magnus University, K. Donelaičio Str. 58, 44248 Kaunas, Lithuania; aidas.perminas@vdu.lt; 4 Turing College, Žalgirio g. 90-D, 09300 Vilnius, Lithuania; lukas@turingcollege.com (L.K.); giedrius.zebrauskas@turingcollege.com (G.Z.)

**Keywords:** e-learning, cognitive abilities, personality, motivation, computer programming

## Abstract

Educational systems around the world encourage students to engage in programming activities, but programming learning is one of the most challenging learning tasks. Thus, it was significant to explore the factors related to programming learning. This study aimed to identify computer programming e-learners’ personality traits, self-reported cognitive abilities and learning motivating factors in comparison with other e-learners. We applied a learning motivating factors questionnaire, the Big Five Inventory—2, and the SRMCA instruments. The sample consisted of 444 e-learners, including 189 computer programming e-learners, the mean age was 25.19 years. It was found that computer programming e-learners demonstrated significantly lower scores of extraversion, and significantly lower scores of motivating factors of individual attitude and expectation, reward and recognition, and punishment. No significant differences were found in the scores of self-reported cognitive abilities between the groups. In the group of computer programming e-learners, extraversion was a significant predictor of individual attitude and expectation; conscientiousness and extraversion were significant predictors of challenging goals; extraversion and agreeableness were significant predictors of clear direction; open-mindedness was a significant predictor of a diminished motivating factor of punishment; negative emotionality was a significant predictor of social pressure and competition; comprehension-knowledge was a significant predictor of individual attitude and expectation; fluid reasoning and comprehension-knowledge were significant predictors of challenging goals; comprehension-knowledge was a significant predictor of clear direction; and visual processing was a significant predictor of social pressure and competition. The SEM analysis demonstrated that personality traits (namely, extraversion, conscientiousness, and reverted negative emotionality) statistically significantly predict learning motivating factors (namely, individual attitude and expectation, and clear direction), but the impact of self-reported cognitive abilities in the model was negligible in both groups of participants and non-participants of e-learning based computer programming courses; χ² (34) = 51.992, *p* = 0.025; CFI = 0.982; TLI = 0.970; NFI = 0.950; RMSEA = 0.051 [0.019–0.078]; SRMR = 0.038. However, as this study applied self-reported measures, we strongly suggest applying neurocognitive methods in future research.

## 1. Introduction

Educational systems worldwide encourage students to engage in programming activities, because learning computer programming improves cognitive skills, including creativity, reasoning, and mathematical skills [1]. Acquiring computer programming skills also ensures large work possibilities, as hiring managers prefer candidates who know programming languages and demonstrate computational literacy [2]. Moreover, programming competences are among the core skills that professionals are expected to possess in the era of rapid technology development [3]. Nevertheless, computer programing is one of the most challenging learning tasks [4], as the dropout rates or failures in programming language courses are very high [5]. Research indicates that the main reasons for this can be learners’ characteristics [6] and learning motivation [4,7,8], not to mention the genetic factors [9], the links between learning and the hippocampal region, which has a key role in cognitive processes [10], the impact of cerebral metabolism [11,12], or neurotransmitters [13], neurotrophic factors [14], and the dynamic interactions between the distributed brain areas and neuronal networks [15,16,17,18,19,20].

### 1.1. Learning Motivation

Learning motivation can be defined as the extent to which persistent efforts are directed toward learning as a goal [21,22]. Motivated learners put in more efforts, are more attentive and more persistent in face of difficulties [23].

Law et al. [4] suggested grouping intrinsic and extrinsic learning motivation factors into several research-based domains. The individual attitude and expectation domain is based on expectancy theory [24], suggesting that motivation is a multiplicative function of three constructs: expectancy (people have different expectations and levels of confidence about what they are capable of doing), instrumentality (the perceptions of individuals whether they will obtain what they desire), and valence (the emotional orientations people hold concerning outcomes or rewards).

The challenging goals domain is based on the assumption that personal goals are essential in determining performance [25,26,27].

The clear direction domain is based on research evidencing that effective learning in higher education is associated with a student’s perception of clear direction [28], with clear direction, learners respond more positively [29].

The reward and recognition domain is based on reinforcement theory, which emphasizes the links between behavior and its consequences [30]. It implies that the anticipation of performance evaluation can affect the motivational direction and task involvement during task performance. These motivational processes may influence subsequent interest in the task, as proper reward and recognition can be a key motivator of learning [31].

The punishment domain is based on the assumption that rewards and punishments and the expectation of punishments motivate some people, yet it also may act as a demotivating factor if too much punishment is applied as the instrument of motivation [30].

The social pressure and competition domain is based on evidence that social forces such as peer pressure and competition also affect learning [32,33,34]. As noted by Law et al., “peer-learning among students in higher education is increasingly a meaningful and important topic for research” [4].

The efficacy domain is based on the assumption that learning efficacy or learning-related self-efficacy refers to what a person believes he or she can perform in a particular learning task and indicates that self-efficacy is related to academic performance [35], as people with a high level of self-efficacy are likely to perform well [36,37,38].

Research indicates that learning motivation is a crucial factor in determining learning outcomes [22,23,39,40]. Even though many authors analyzed learning motivation in computer programming learning, the ambiguous findings [2,41,42] indicate that computer learners’ learning motivation is under-researched.

### 1.2. Cognitive Abilities

The Cattell–Horn–Carroll (CHC) theory of cognitive abilities merged Cattell and Horn’s [43,44,45] Gf-Gc theory with Carroll’s [46,47] three-stratum theory and is viewed as the most validated theory of cognitive abilities currently available [48,49,50,51], supported by developmental, neurocognitive, and heritability evidence [52]. The CHC theory outlines 16 cognitive abilities, which subsume more than 70 narrow abilities [53]. Most intelligence batteries that are used, including the Wechsler Intelligence Scale (WISC-III) [54], the WASI-II [55], the Stanford–Binet (SB-IV) [56], and the Kaufman Brief Intelligence Test (KBIT) [57], among others, with some regularity subscribe either explicitly or implicitly to the CHC theory [50,58].

There is a debate over the question of whether cognitive abilities can potentially be assessed via self-report measures [58]. Self-report measures are used to assess a wide variety of psychological constructs and are valued for low cost and easy administration, the capability to assess large numbers of individuals simultaneously, and a less anxiety-inducing assessment format [59,60]. Research on the validity of self-reports of cognitive abilities suggests that individuals have only limited insight [61,62]. However, some studies have demonstrated high correlations between the self-report of specific cognitive ability areas and performance [63,64] or indicated that individuals can differentiate between distinct cognitive abilities when providing self-ratings [65,66].

In 2014, Jacobs et al. [67] developed the self-report measure of cognitive abilities (SRMCA) as an indicator of the level of cognitive functioning in three key CHC broad ability areas: fluid reasoning (Gf), comprehension-knowledge (Gc), and visual processing (Gv). The fluid reasoning cognitive ability domain represents an individual’s ability to reason, form concepts and solve problems that often involve unfamiliar information or procedures. This ability area influences one’s ability to draw inferences, solve abstract problems, create solutions to problems, and think conceptually. The comprehension-knowledge cognitive ability domain represents the breadth and depth of an individual’s store of general information and acquired verbal knowledge. This ability area influences the extent of one’s vocabulary and the ability to answer factual questions and comprehend written and spoken language. The visual processing cognitive ability domain represents an individual’s ability to perceive, analyze, synthesize, and think with visual patterns, including storing and recalling visual representations. This ability area influences one’s ability to perform tasks such as assembling puzzles, using patterns and designs in art and geography, sensing orientation, and reading maps, charts, graphs, and blueprints [67].

However, computer programming learners’ cognitive abilities are under-researched, even though some researchers analyzed programmers’ creativity and analytical problem-solving skills [68], and it was concluded that competent programmers are characterized as possessing outstanding cognitive abilities [6,69].

### 1.3. Personality Traits

Individual differences in people’s patterns of thinking, feeling, and behaving can be summarized in the Big Five personality domains, namely, extraversion, agreeableness, conscientiousness, neuroticism, and openness to experience, which also cover 15 facets [70]. These Big Five personality domains were extensively analyzed applying the NEO personality inventory [71] or the Big Five Inventory-2 [70] in various contexts, including work, education, or sports. Research demonstrated that neuroticism is related to diminished self-efficacy [72] and higher work-family conflict, while conscientiousness is related to better self-regulation [73].

Many studies analyzed the relationship between personality traits and learning motivation [74,75,76]. Even several decades ago, in 1989, Heaven demonstrated that high school students’ achievement motivation is positively related to extraversion and negatively related to impulsiveness and psychoticism [77]. Afterward, research indicated that conscientiousness and extraversion of learners are related to learning achievements [78]. Other studies demonstrated that conscientiousness, extraversion, and openness strongly relate to learning goal orientation, while neuroticism and low extraversion relate to a fear of failure [79]. It was also revealed that only extraversion, conscientiousness, and openness significantly affect students’ level of intrinsic motivation to learn [80]. Next, it was found that neuroticism and extroversion are positively related to extrinsic academic motivation, and conscientiousness is positively related to intrinsic academic motivation [81]. Recent research reported that conscientiousness and m-learning readiness are critical antecedents of intrinsic motivation and extrinsic motivation [82].

To sum, many studies confirmed that learners’ personality traits have a significant influence on learning motivation [83,84], but the findings are ambiguous. Some studies revealed a positive relationship between neuroticism and intrinsic academic motivation, and between extroversion and conscientiousness and extrinsic academic motivation [85], or a positive relationship between conscientiousness and openness to experience and intrinsic learning motivation, or between extroversion and neuroticism and extrinsic learning motivation [81]. Thus, the links between learning motivating factors and personality are not explored sufficiently.

Likewise, computer programming learners’ personality traits are also under-researched, even though there have been many attempts to identify personality traits linked to programming performance [86,87,88,89,90]. A meta-analysis on 19 independent samples (total *n* = 1695) showed that programming aptitude does not associate with disagreeableness or neuroticism but associates with conscientiousness, openness, and introversion, and these traits explain incremental variance components beyond general mental abilities [89].

In order to fill the gap, this study primarily aimed to identify computer programming e-learners’ personality traits, self-reported cognitive abilities and learning motivating factors. Based on the previous research, we hypothesized that:

**Hypothesis** **1** **(H1).** *Computer programming e-learners differ in their personality traits from other e-learners*;

**Hypothesis** **2** **(H2).** *Computer programming e-learners differ in their self-reported cognitive abilities from other e-learners*;

**Hypothesis** **3** **(H3).** *Computer programming e-learners differ in their learning motivating factors from other e-learners*;

**Hypothesis** **4** **(H4).** *Personality traits predict learning motivating factors in both groups of participants and non-participants of computer programming courses*;

**Hypothesis** **5** **(H5).** *Self-reported cognitive abilities predict learning motivating factors of participants and non-participants of computer programming courses*;

**Hypothesis** **6** **(H6).** *There exist associations between learning motivating factors, personality traits, and self-reported cognitive abilities, but they differ between participants and non-participants of e-learning based computer programming courses*.

## 2. Materials and Methods

### 2.1. Sample

In the full sample of 481 participants, a total of 481 participants had no missing data. Because the number of cases with missing values was small, we used the listwise deletion of cases with missing values. Therefore, all analyses were conducted using a sample of 481 individuals. The study’s subjects comprised of 33.3% males (*n* = 160) and 66.7% females (*n* = 321). The respondents’ mean age was 25.07 years (SD = 8.248, 95% CI = 24.33, 25.81, age range =18 to 57 years). A total of 189 (39.3%) of participants studied in e-learning based computer programming courses organized by Turing College, while the rest 292 (60.7%) studied in other Lithuanian Universities. Participation in the study was voluntary, and the participants did not receive any compensation. The procedure was administered online at psytest.online and followed the General Data Protection Regulation (GDPR) guidelines and the requirements of the Helsinki Declaration.

### 2.2. Instruments

This study applied three instruments, the translated Lithuanian version of the learning motivating factors questionnaire [4,7], the translated Lithuanian version of the self-report measure of cognitive abilities (SRMCA) [67], and the translated Lithuanian version of the Big Five Inventory-2 (BFI-2) [70]. To ensure that the Lithuanian items correspond as closely as possible to the English items, the original items of both instruments were translated into Lithuanian and back-translated.

The Self-Report Measure of Cognitive Abilities (SRMCA). To assess self-reported cognitive abilities, we applied the self-report measure of cognitive abilities (SRMCA), developed by Jacobs et al. [67]. The SRMCA is a 25-item scale that evaluates self-reported cognitive functioning in three key CHC broad ability areas: fluid reasoning (Gf), comprehension-knowledge (Gc), and visual processing (Gv). The statements ask to rate how easy or difficult a person usually finds it to perform specific tasks, and when responding to each item, respondents compare themselves to most people of their age; “compared to most people my age, I usually find tasks requiring me to….” The response pattern followed a Likert scale ranging from 1 (extremely difficult) to 7 (extremely easy). Cronbach’s alpha for the scale in this study was 0.897.

The Learning Motivating Factors Questionnaire. To assess learning motivation, we applied the learning motivating factors questionnaire, developed by Law et al. [4,7]. This 19-items questionnaire measures factors that positively motivate learning and covers several motivational variables listed further. The individual attitude and expectation subscale measures a student’s attitude and expectations towards learning. The challenging goals subscale measures perceived challenging goals in learning. The clear direction subscale measures perceived specified direction in learning. The reward and recognition subscale measures perceived positive reinforcements such as reward, appreciation, and encouragement. The punishment subscale measures the perceived negative reinforcement due to punishment. The social pressure and competition subscale measures perceived forces of pressure and competition from peers. The response pattern followed a 6-point Likert scale, ranging from 1 (disagree very much) to 6 (agree very much). Cronbach’s alpha for the scale in this study was 0.869.

The Big Five Inventory–2 (BFI-2). Personality traits were measured by the Big Five Inventory-2 (BFI-2), which uses 60 items to hierarchically assess the Big Five personality domains; namely, extraversion, agreeableness, conscientiousness, neuroticism, and openness to experience, and 15 more-specific facet traits [70]. The BFI-2 items are short, descriptive phrases with the shared item: ‘‘I am someone who...” (e.g., ‘‘is outgoing, sociable,” ‘‘tends to be quiet”). Respondents rated each item using a 5-point Likert scale ranging from 1 (disagree strongly) to 5 (agree strongly). Cronbach’s alpha for the scale in this study was 0.786.

### 2.3. Statistical Analysis

For the data analysis, we used SPSS v.26.0. The structural equation modeling (SEM) was conducted using AMOS v.26.0 and JASP v.0.14.1.0. The model fit was evaluated based on the CFI (comparative fit index), the normed fit index (NFI), the Tucker—Lewis coefficient (TLI), the root mean square error of approximation (RMSEA), and the standardized root mean square residual (SRMR), whereas the χ^2^ was used for descriptive purposes only because it is susceptible to the sample size [91]. The values higher than 0.90 for CFI, NFI, TLI, and values lower than 0.08 for RMSEA and SRMR were considered as indicative of a good fit [92]. In this research, we considered *p*-values less than 0.05 to be statistically significant [93].

The Shapiro—Wilk test showed the departure from normality for the variables of individual attitude and expectation, W (406) = 0.960, *p* < 0.001; challenging goals, W (406) = 0.965, *p* < 0.001; clear direction, W (406) = 0.936, *p* < 0.001; reward and recognition, W (406) = 0.934, *p* < 0.001; punishment, W (406) = 0.967, *p* < 0.001; social pressure and competition, W (406) = 0.984, *p* < 0.001; fluid reasoning, W (455) = 0.981, *p* < 0.001; comprehension-knowledge, W (455) = 0.978, *p* < 0.001; visual processing, W (455) = 0.969, *p* < 0.001; agreeableness, W (480) = 0.991, *p* = 0.005; conscientiousness, W (480) = 0.989, *p* = 0.001; negative emotionality, W (480) = 0.993, *p* = 0.018; and open-mindedness, W (480) = 0.988, *p* < 0.001. However, the data were normally distributed for the following variables: extraversion W (480) = 0.994, *p* = 0.063.

Similarly, Kolmogorov–Smirnov test showed that data were non-normally distributed for the variables of individual attitude and expectation, D (406) = 0.113, *p* < 0.001; challenging goals, D (406) = 0.094, *p* < 0.001; clear direction, D (406) = 0.139, *p* < 0.001; reward and recognition, D (406) = 0.142, *p* < 0.001; punishment, D (406) = 0.086, *p* < 0.001; social pressure and competition, D (406) = 0.067, *p* < 0.001; fluid reasoning, D (455) = 0.060, *p* < 0.001; comprehension-knowledge, D (455) = 0.081, *p* < 0.001; visual processing, D (455) = 0.111, *p* < 0.001; extraversion, D (480) = 0.043, *p* = 0.033; agreeableness, D (480) = 0.059, *p* < 0.001; conscientiousness, D (480) = 0.054, *p* = 0.002, negative emotionality, D (480) = 0.051, *p* = 0.004; and open-mindedness, D (480) = 0.064, *p* < 0.001.

The distribution was moderately skewed: individual attitude and expectation skewness = −0.494 (SE = 0.121), kurtosis = −0.028 (SE = 0.242); challenging goals skewness = −0.435 (SE = 0.121), kurtosis = −0.190 (SE = 0.242); clear direction skewness = −0.435 (SE = 0.121), kurtosis = −0.176 (SE = 0.242); reward and recognition skewness = −0.661 (SE = 0.121), kurtosis = −0.045 (SE = 0.242); punishment skewness = −0.038 (SE = 0.121), kurtosis = −0.642 (SE = 0.242); social pressure and competition skewness = −0.030 (SE = 0.121), kurtosis = −0.500 (SE = 0.242); fluid reasoning skewness = −0.369 (SE = 0.114), kurtosis = −0.375 (SE = 0.228); comprehension-knowledge skewness = −0.326 (SE = 0.114), kurtosis = −0.551 (SE = 0.228); visual processing skewness = −0.461 (SE = 0.114), kurtosis = −0.533 (SE = 0.228); extraversion skewness = −0.158 (SE = 0.111), kurtosis = −0.139 (SE = 0.222); agreeableness skewness = −0.248 (SE = 0.111), kurtosis = −0.212 (SE = 0.222); conscientiousness skewness = −0.283 (SE = 0.111), kurtosis = −0.185 (SE = 0.222); negative emotionality = 0.094 (SE = 0.111), kurtosis = −0.484 (SE = 0.222); open-mindedness skewness = −0.279 (SE = 0.111), kurtosis = −0.266 (SE = 0.222).

Therefore, we conducted a square root transformation (SQRT) of significantly negatively skewed variables to create normally distributed variables and conduct the CFA analyses.

## 3. Results

Descriptive statistics of the learning motivating factors questionnaire subscales in this study are reported in Table 1.

Means, standard deviations, and correlations between the BFI-2 subscales in this study are reported in Table 2.

Means, standard deviations, and correlations between the SRMCA subscales in this study are reported in Table 3.

To test Hypothesis 1 (H1), assuming that computer programming e-learners differ in their personality traits from other e-learners, we conducted the independent samples *t*-test. The results are displayed in Table 4.

The *t*-test analysis revealed significant differences between groups: non-participants of computer programming e-learning courses demonstrated higher scores (M = 3.36, SD = 0.59) of extraversion than computer programming e-learning courses (M = 3.22, SD = 0.61), *p* = 0.015.

Furthermore, to test Hypothesis 2 (H2), which presumed that computer programming e-learners differ in their self-reported cognitive abilities from other e-learners, we also conducted the independent samples’ *t*-test (Table 5). The *t*-test analysis revealed no statistically significant differences between the groups.

Furthermore, to test Hypothesis 3 (H3), assuming that computer programming e-learners differ in their learning motivating factors from other e-learners, we conducted a *t*-test. The results of the test are displayed in Table 6.

The *t*-test analysis revealed significant differences between groups of participants and non-participants of e-learning based computer programming courses in the scores of individual attitude and expectation, reward and recognition, and punishment. Non-participants of e-learning based computer programming courses demonstrated higher scores in individual attitude and expectation (M = 4.82, SD = 0.81) than computer programming e-learners (M = 4.58, SD = 0.91), *p* = 0.004. Non-participants also demonstrated higher scores in reward and recognition (M = 4.97, SD = 0.87) than computer programming e-learners (M = 4.70, SD = 0.92), *p* = 0.002. Finally, non-participants demonstrated higher scores in punishment (M = 3.64, SD = 1.35) than those who participated in e-learning based computer programming courses (M = 3.24, SD = 1.28), *p* = 0.002.

Furthermore, to test Hypothesis 4 (H4), presuming that personality traits predict learning motivating factors in both groups of participants and non-participants of e-learning based computer programming courses, we conducted a multiple linear regression (forward method) analysis. Statistically significant results in the group of computer programming e-learners are displayed in Table 7.

In the group of computer programming e-learners, a significant regression equation was found concerning the motivational factor of individual attitude and expectation, F (1, 180) = 21.373, *p* < 0.001, with R^2^ = 0.106. Predicted individual attitude and expectation was equal to 3.039 + 0.482 (extraversion) points. Individual attitude and expectation increased 0.482 points for each extraversion point (*p* < 0.001). Thus, extraversion contributed significantly to the model and was a significant predictor of individual attitude and expectation of computer programming e-learners.

A significant regression equation was also found regarding the motivational factor of challenging goals. In model 1, the dependent variable was challenging goals, and the predictor was conscientiousness, F (2, 179) = 50.661, *p* < 0.001, with R^2^ = 0.220. The predicted challenging goals factor was equal to 1.610 + 0.810 (conscientiousness). Challenging goals increased + 0.810 for each conscientiousness point. In model 2, the dependent variable was the individual attitude and expectation, and the predictor was conscientiousness and extraversion, F (2, 179) = 30.600, *p* = 0.004, with R^2^ = 0.255. The predicted challenging goals factor was equal to 1.026 + 0.645 (conscientiousness) + 0.357 (extraversion). Challenging goals increased + 0.645 for each conscientiousness (*p* < 0.001) point and + 0.357 for each extraversion (*p* = 0.004) point. Thus, conscientiousness and extraversion were significant predictors of challenging goals of computer programming e-learners.

Next, a significant regression equation was found in relation to a motivational factor of clear direction. In model 1, the dependent variable was clear direction, and the predictor was extraversion, F (2, 179) = 22.173, *p* < 0.001, with R^2^ = 0.110. The predicted clear direction was equal to 3.527 + 0.424 (extraversion). Clear direction increased + 0.424 for each extraversion point. In model 2, the dependent variable was clear direction, and the predictor was extraversion and agreeableness, F (2, 179) = 16.101, *p* = 0.003, with R^2^ = 0.152. The predicted clear direction was equal to 2.452 + 0.391 (extraversion) + 0.330 (agreeableness). Clear direction increased + 0.391 for each extraversion (*p* < 0.001) point and + 0.330 for each agreeableness (*p* = 0.004) point. So, extraversion and agreeableness were significant predictors of clear direction of computer programming e-learners.

Then, a significant regression equation was found concerning motivational factor of punishment, F (1, 180) = 4.400, *p* = 0.037, with R^2^ = 0.024. The predicted punishment was equal to 4.838 − 0.439 (open-mindedness) points. Punishment decreased 0.439 points for each open-mindedness point. Therefore, open-mindedness (*p* = 0.037) was a significant predictor of the diminished motivational factor of punishment in the group of participants of e-learning based computer programming courses.

Finally, a significant regression equation was found regarding the motivational factor of social pressure and competition, F (1, 180) = 4.810, *p* = 0.030. The predicted social pressure and competition was equal to 4.048 − 0.227 (negative emotionality) points. Social pressure and competition decreased 0.227 points for each negative emotionality point. This means that negative emotionality (*p* = 0.030) was a significant predictor of social pressure and competition of computer programming e-learners.

Next, a multiple linear regression (forward method) was likewise calculated to predict learning motivating factors based on personality traits in the group of respondents, who did not participate in e-learning based computer programming courses. The results of the multiple linear regression models (the dependent variables are learning motivating factors, and the predictors are personality traits), are displayed in Table 8.

In the group of non-participants of e-learning based computer programming courses, a significant regression equation was found regarding the motivational factor of individual attitude and expectation, F (1, 220) = 9.866, *p* = 0.002, with R^2^ = 0.043. The predicted individual attitude and expectation was equal to 3.898 + 0.276 (conscientiousness) points. Individual attitude and expectation increased 0.276 points for each conscientiousness point. Thus, conscientiousness (*p* = 0.002) was a significant predictor of individual attitude and expectation of non-participants of e-learning based computer programming courses.

Next, a significant regression equation was found concerning the motivational factor of challenging goals. In model 1, the dependent variable was challenging goals, and the predictor was conscientiousness, F (2, 219) = 16.021, *p* < 0.001, with R^2^ = 0.068. The predicted challenging goals factor was equal to 2.687 + 0.483 (conscientiousness). Challenging goals increased + 0.483 points for each conscientiousness (*p* < 0.001) point. In model 2, the dependent variable was challenging goals, and the predictors were conscientiousness and negative emotionality, F (2, 219) = 11.861, *p* < 0.001, with R^2^ = 0.098. The predicted challenging goals factor was equal to 3.837 + 0.400 (conscientiousness) − 0.283 (negative emotionality). Challenging goals increased + 0.400 for each conscientiousness (*p* = 0.001) point and decreased 0.283 for each negative emotionality (*p* = 0.008) point. Hence, conscientiousness and negative emotionality were significant predictors of the motivational factor of challenging goals in the group of respondents who did not participate in e-learning based computer programming courses.

A significant regression equation was found in regard to clear direction. In model 1, the dependent variable was clear direction, and the predictor was extraversion, F (2, 219) = 19.116, *p* < 0.001, with R^2^ = 0.080. Predicted clear direction was equal to 3.694 + 0.395 (extraversion). Clear direction increased + 0.395 for each extraversion point. In model 2, the dependent variable was clear direction, and the predictors were extraversion and agreeableness, F (2, 219) = 12.714, *p* < 0.001, with R^2^ = 0.104. Predicted clear direction was equal to 3.286 + 0.300 (extraversion) + 0.217 (agreeableness). Clear direction increased + 0.300 for each extraversion (*p* = 0.002) point and + 0.217 for each agreeableness (*p* = 0.016) point. Thus, extraversion and agreeableness were significant predictors of clear direction in group of other e-learners who did not participate in e-learning based computer programming courses.

Lastly, a significant regression equation was found in regard to the reward and recognition motivational factor, F (1, 220) = 9.011, *p* < 0.001, with R^2^ = 0.039. The predicted reward and recognition was equal to 4.019 + 0.284 (conscientiousness) points. Reward and recognition increased 0.284 points for each conscientiousness (*p* = 0.003) point. This means that conscientiousness significantly predicted the motivational factor of reward and recognition in the group of non-participants of e-learning based computer programming courses.

Furthermore, to test Hypothesis 5 (H5), presuming that self-reported cognitive abilities predict learning motivating factors of computer programming e-learners, we conducted a multiple linear regression (forward method), using learning motivating factors as the criterion, and self-reported cognitive abilities as the predictors. Statistically significant results in the group of computer programming e-learners are displayed in Table 9.

In the group of respondents participating in e-learning based computer programming courses, a significant regression equation was found regarding the motivational factor of individual attitude and expectation, F (1, 181) = 8.060, *p* = 0.005, with R^2^ = 0.043. The predicted individual attitude and expectation was equal to 3.577 + 0.197 (comprehension-knowledge) points. Individual attitude and expectation increased 0.197 points for each comprehension-knowledge point (*p* = 0.005). Thus, comprehension-knowledge was a significant predictor of individual attitude and expectation of computer programming e-learners.

Next, a significant regression equation was found concerning the motivational factor of challenging goals. In model 1, the dependent variable was challenging goals, and the predictor was fluid reasoning, F (2, 180) = 41.361, *p* < 0.001, with R^2^ = 0.186. The predicted challenging goals factor was equal to 1.941 + 0.477 (fluid reasoning). The Challenging goals increased + 0.477 for each fluid reasoning (*p* = 0.002) point. In model 2, the dependent variable was challenging goals, and the predictors were fluid reasoning and comprehension-knowledge, F (2, 180) = 26.194, *p* < 0.001, with R^2^ = 0.225. The predicted challenging goals was equal to 1.428 + 0.299 (fluid reasoning *p* = 0.002) + 0.279 (comprehension-knowledge *p* = 0.003). Challenging goals increased + 0.299 for each fluid reasoning point and + 0.279 for each comprehension-knowledge point. Thus, fluid reasoning and comprehension-knowledge were significant predictors of challenging goals of computer programming e-learners.

Furthermore, a significant regression equation was found in regard to the clear direction motivational factor, F (1, 181) = 16.734, *p* < 0.001, with R^2^ = 0.085. The predicted clear direction was equal to 3.673 + 0.240 (comprehension-knowledge) points. Clear direction increased 0.240 points for each comprehension-knowledge (*p* < 0.001) point. Hence, comprehension-knowledge was a significant predictor of clear direction in the group of computer programming e-learners.

Finally, a significant regression equation was found concerning the motivational factor of social pressure and competition, F (1, 181) = 6.212, *p* = 0.014, with R^2^ = 0.033. The predicted social pressure and competition was equal to 1.951 + 0.256 (visual processing) points. Social pressure and competition increased 0.256 points for each visual processing (*p* = 0.014) point, which means that visual processing was a significant predictor of social pressure and competition of participants of e-learning based computer programming courses.

Then, we conducted a multiple linear regression (forward method) in the group of non-participants of computer programming e-learning courses, using learning motivating factors as the criterion, and self-reported cognitive abilities as the predictors. The results are displayed in Table 10.

In the group of respondents who did not participate in e-learning based computer programming courses, a significant regression equation was found concerning the motivational factor of individual attitude and expectation, F (1, 220) = 8.739, *p* = 0.003, with R^2^ = 0.038. The predicted individual attitude and expectation was equal to 3.961 + 0.168 (fluid reasoning) points. Individual attitude and expectation increased 0.168 points for each fluid reasoning (*p* = 0.003) point. Thus, fluid reasoning was a significant predictor of individual attitude and expectation of other e-learners who did not participate in computer programming courses.

Next, a significant regression equation was found in regard to the motivational factor of challenging goals, F (1, 220) = 12.212, *p* = 0.001, with R^2^ = 0.053. The predicted challenging goals was equal to 2.899 + 0.273 (fluid reasoning) points. Challenging goals increased 0.273 points for each fluid reasoning (*p* = 0.001) point. Therefore, fluid reasoning was a significant predictor of challenging goals of non-participants of e-learning based computer programming courses.

Likewise, a significant regression equation was found concerning the motivational factor of clear direction, F (1, 220) = 5.491, *p* = 0.020, with R^2^ = 0.024. The predicted clear direction was equal to 4.243 + 0.146 (visual processing) points. Clear direction increased 0.146 points for each visual processing (*p* = 0.020) point. So, visual processing was a significant predictor of clear direction in the group of other e-learners who did not participate in computer programming courses.

After all, a significant regression equation was found regarding the reward and recognition motivational factor, F (1, 220) = 10.664, *p* = 0.001, with R^2^ = 0.046. The predicted reward and recognition was equal to 3.750 + 0.225 (visual processing) points. Reward and recognition increased 0.225 points for each visual processing (*p* = 0.001) point. So, visual processing was a significant predictor of reward and recognition in the group of non-participants of e-learning based computer programming courses.

**Figure 1:**
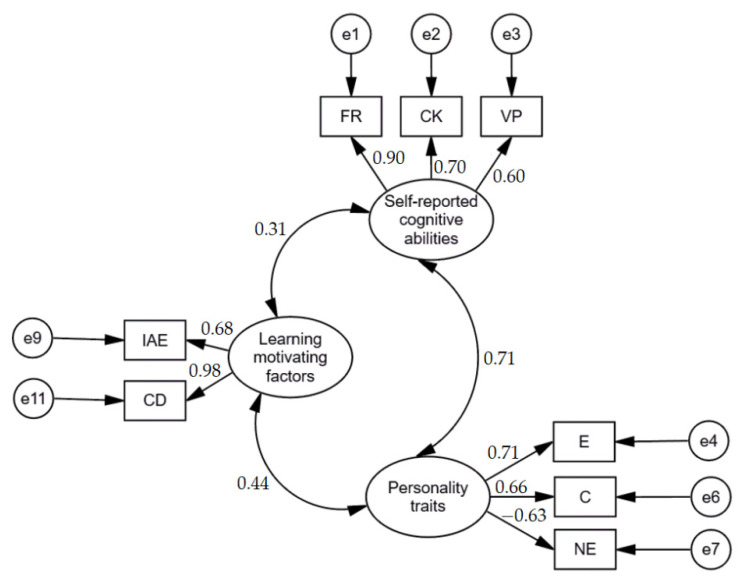
Standardized results of model on associations between learning motivating factors, self-reported cognitive abilities, and personality traits in group of computer programming e-learners. IAE: individual attitude and expectation; CD: clear direction; E: extraversion; C: conscientiousness; NE: negative emotionality; FR: fluid reasoning; CK: comprehension-knowledge; VP: visual processing.

Furthermore, to test Hypothesis 6 (H6), which assumed that there exist associations between learning motivating factors, personality traits, and self-reported cognitive abilities, but they differ between participants and non-participants of e-learning based computer programming courses, we conducted several SEM analyses. Firstly, based on previous results, we created a model on associations between learning motivating factors, self-reported cognitive abilities, and personality traits in the group of computer programming e-learners. Standardized results of the model are presented in Figure 1. The model fit was evaluated based on the CFI (Comparative Fit Index), the normed fit index (NFI), the Tucker–Lewis coefficient (TLI), and the root mean square error of approximation (RMSEA), whereas the χ^2^ was used for descriptive purposes only [91]. As mentioned above, the values higher than 0.90 for CFI, NFI, and TLI, and the values lower than 0.08 for RMSEA are considered as indicative of a good fit [92]. Findings revealed that the fit of the model was good, χ^2^ = 34.707; df = 17; NFI = 0.930; TLI = 0.919; CFI = 0.962; RMSEA = 0.074 [.038 – 0.110].

**Figure 2:**
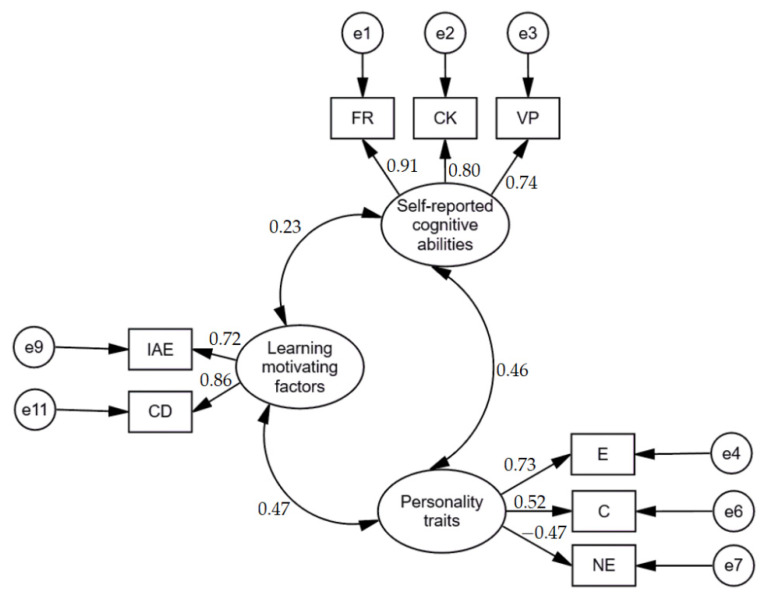
Standardized results of model on associations between learning motivating factors, self-reported cognitive abilities, and personality traits in group of non-participants of computer programming e-learning based courses. IAE: individual attitude and expectation; CD: clear direction; E: extraversion; C: conscientiousness; NE: negative emotionality; FR: fluid reasoning; CK: Comprehension-Knowledge; VP: visual processing.

Then, we created a model on associations between learning motivating factors, self-reported cognitive abilities, and personality traits in the group of non-participants of computer programming e-learning based courses. Standardized results of the model are presented in Figure 2. Findings revealed that the fit of the model was good, χ^2^ = 22.033; df = 17; NFI = 0.966; TLI = 0.983; CFI = 0.992; RMSEA = 0.032 [0.000–0.066].

**Figure 3:**
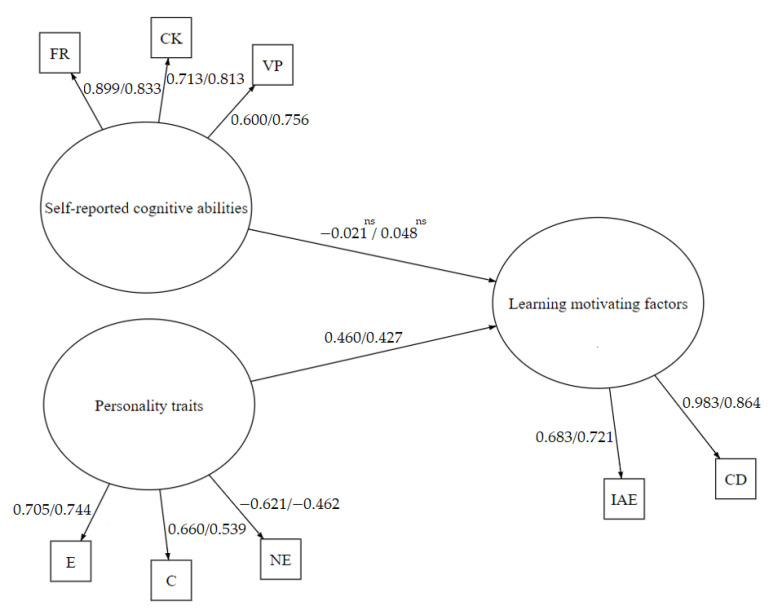
Standardized results of model on associations between learning motivating factors, self-reported cognitive abilities, and personality traits in both groups of participants and non-participants of computer programming e-learning based courses. IAE: individual attitude and expectation; CD: clear direction; E: extraversion; C: conscientiousness; NE: negative emotionality; FR: fluid reasoning; CK: comprehension-knowledge; VP: visual processing; ns: non-significant.

Afterwards, based on previous analyses, we examined an alternative model on the associations between learning motivating factors, self-reported cognitive abilities, and personality traits in both groups of participants and non-participants of computer programming e-learning based courses. Standardized results of the model are presented in Figure 3.

The model fit was evaluated based on the comparative fit index (CFI), the normed fit index (NFI), the Tucker–Lewis coefficient (TLI), root mean square error of approximation (RMSEA), and standardized root mean square residual (SRMR). The values higher than 0.90 for CFI, NFI, and TLI, and values lower than 0.08 for RMSEA and SRMR were considered as indicative of a good fit [92]. The results showed that model fit was good χ² (34) = 51.992, *p* = 0.025; CFI = 0.982; TLI = 0.970; NFI = 0.950; RMSEA = 0.051 [0.019–0.078]; SRMR = 0.038. Scalar estimates of the model in both groups are displayed in Table 11.

To sum, the SEM analysis showed that personality traits (namely, extraversion, conscientiousness, and reverted negative emotionality) statistically significantly predict learning motivating factors (namely, individual attitude and expectation, and clear direction), and the impact of self-reported cognitive abilities in the model was negligible in both groups of participants and non-participants of e-learning based computer programming courses.

## 4. Discussion

This study was the first to explore computer programming e-learners’ personality traits, self-reported cognitive abilities, and learning motivating factors compared with other e-learners. The assessment of learning motivating factors was based on a model of extrinsic and intrinsic learning motivating factors, developed by Law et al. [4]. It was significant to identify the specifics in computer programing learning because educational systems around the world encourage students to engage in programming activities, which improve cognitive skills, including creativity, reasoning, and mathematical skills [1]. Furthermore, acquiring computer programming skills also ensures large work possibilities, as hiring managers prefer candidates who know programming languages [2,3]. However, computer programming learning is one of the most challenging tasks, indicating high dropout rates or failures [5]. Thus, it was significant to explore the factors that researchers point out as related to programming learning outcomes [6,8,68,69], and this study aimed to identify computer programming e-learners’ personality traits, self-reported cognitive abilities and learning motivating factors.

### 4.1. Computer Programming E-Learners Demonstrated Significantly Lower Extraversion Scores Than Non-Participants of E-Learning Based Computer Programming Courses

In this study, we assumed that computer programming e-learners differ in their personality traits from other e-learners. Thus, we compared the scores of personality traits based on the Big Five Inventory-2 categories in the groups of participants and non-participants of e-learning based computer programming courses. The results partially confirmed this hypothesis: a *t*-test analysis revealed significant differences between the groups: non-participants of computer programming e-learning courses demonstrated higher scores of personality trait extraversion than participants of computer programming e-learning courses. However, there were no significant differences in the scores of agreeableness, conscientiousness, neuroticism, and openness to experience. These results align with some previous research suggesting that computer programming e-learners might demonstrate lower scores in extraversion [86,87,88,89,90]. However, it is unclear why no significant differences were found in other traits, namely, conscientiousness or open-mindedness, as indicated by some previous research [89].

Interestingly, a magnetic resonance imaging (MRI) study by Gardini et al. [94] indicated that individual differences in the central personality dimensions might reflect structural variance in specific brain areas. Researchers found that personality dimension novelty seeking correlated with grey matter volume in frontal and posterior cingulate regions, harm avoidance showed a negative correlation with grey matter volume in orbitofrontal, occipital and parietal structures, and reward dependence was negatively correlated with grey matter volume in the caudate nucleus and the rectal frontal gyrus. Next, personality dimension persistence was positively correlated with grey matter volume in the precuneus, paracentral lobule, and parahippocampal gyrus [94]. Furthermore, Kabbara et al. [95] found a significant relationship between neuroticism and the dynamic variability of the temporal lobe regions. They highlighted the importance of tracking the dynamics of functional brain networks to improve understanding of the neural substrates of personality [95]. Furthermore, in a study by Lewis et al. [96], conscientiousness trait scores were positively related to brain cortical thickness in a range of regions, including the bilateral parahippocampal gyrus, bilateral fusiform gyrus, left cingulate gyrus, right medial orbitofrontal cortex, and the left dorsomedial prefrontal cortex [96]. Concerning various findings [94,95,96,97,98,99], it can be suggested that computer learners’ personalities need further investigation, especially in establishing links to computer programming e-learners’ brain regions.

### 4.2. No Significant Differences Were Found in the Scores of Self-Reported Cognitive Abilities between the Groups of Participants and Non-Participants of E-Learning Based Computer Programming Courses

Furthermore, we presumed that computer programming e-learners differ in their cognitive abilities from other e-learners. Thus, we compared the scores of self-reported cognitive abilities based on the SRMCA, which indicates the level of cognitive functioning in three key ability areas: fluid reasoning, comprehension-knowledge, and visual processing [67]. The results did not confirm this hypothesis: a *t*-test analysis revealed no statistically significant differences in self-reported cognitive abilities between the groups of participants and non-participants of e-learning based computer programming courses. These findings contradict previous research indicating the excellent cognitive skills of computer programmers, including analytical, problem-solving, and mathematical skills [6,68,69]. These results might point to some methodological issues. This study was based on self-reported measures, and the possible significance of objective, including neurocognitive, indicators was omitted. Thus, even though the authors of the SRMCA indicate that the instrument measures the level of cognitive functioning in three key ability areas of fluid reasoning, comprehension-knowledge, and visual processing [67], it can be presumed that the SRMCA measures only self-efficacy, related to these areas. Therefore, the results of this study should be taken with caution, and we strongly suggest applying neurocognitive methods in future research.

Interestingly, a study by Breukelaar et al. [100] provided evidence that changes in the functional organization of the cognitive control brain network occur despite the absence of neurodevelopment, aging, or targeted cognitive training effects and can modulate cognitive performance in early to mid-adulthood. Comprehensive research by Oschwald et al. [101] provided guidance for future researchers and demonstrated that despite several studies reporting correlations between specific brain regions and specific cognitive abilities (e.g., between structures of the medial temporal lobe and episodic memory), the number of studies presenting converging evidence is small. Concerning these and other studies [100,101,102,103,104,105,106], it can be concluded that computer learners’ cognitive abilities need further investigation, especially in establishing precise links to learners’ structural and functional brain properties.

### 4.3. Computer Programming E-Learners Demonstrated Significantly Lower Scores of Motivating Factors of Individual Attitude and Expectation, Reward and Recognition, and Punishment

Next, we assumed that computer programming e-learners differ in their learning motivating factors from other e-learners. Therefore, we compared the scores of learning motivating factors in the groups of participants and non-participants of e-learning based computer programming courses. The results partially confirmed this hypothesis. A *t*-test analysis revealed significant differences between the groups of participants and non-participants of e-learning based computer programming courses in the scores of individual attitude and expectation, reward and recognition, and punishment. Non-participants of e-learning based computer programming courses demonstrated higher scores in individual attitude and expectation in comparison with computer programming e-learners. Non-participants also demonstrated higher scores in reward and recognition than computer programming e-learners. Finally, non-participants demonstrated higher scores in punishment than those who participated in e-learning based computer programming courses. However, no significant differences were found in a clear direction, challenging goals, or social pressure and competition in the groups of participants and non-participants of e-learning based computer programming courses. Previous research [2,42] can moderately support these findings, but it is unclear why no significant differences were found in clear direction and challenging goals between computer programming and other e-learners [41].

### 4.4. Personality Traits Predict Learning Motivating Factors in Both Groups of Participants and Non-Participants of Computer Programming Courses

Next, we presumed that personality traits predict learning motivating factors in both groups of participants and non-participants of computer programming courses. Thus, we conducted a multiple linear regression using learning motivating factors as the criterion and personality traits as predictors in groups of respondents participating and not participating in e-learning based computer programming courses. The results partially confirmed this hypothesis. It showed that in the group of respondents participating in computer programming courses, extraversion was a significant predictor of individual attitude and expectation; conscientiousness and extraversion were significant predictors of challenging goals; extraversion and agreeableness were significant predictors of clear direction; open-mindedness was a significant predictor of diminished motivational factor of punishment; negative emotionality was a significant predictor of social pressure and competition. On the other hand, in the group of non-participants of e-learning based computer programming courses, conscientiousness was a significant predictor of individual attitude and expectation; conscientiousness and negative emotionality were significant predictors of challenging goals; extraversion and agreeableness were significant predictors of clear direction; conscientiousness significantly predicted reward and recognition. These results to some extent are in line with previous studies which analyzed the links between personality traits and learning motivation [74,75,76,77]. The findings support previous reports indicating that conscientiousness, extraversion, and openness significantly affect students’ level of intrinsic motivation to learn [80], and that these personality traits relate to learning goal orientation, while neuroticism and low extraversion relates to a fear of failure [78,79]. This study also aligns with previous findings, indicating that conscientiousness is positively related to intrinsic academic motivation [81,85], but it does not support previous findings that neuroticism and extroversion are positively related to extrinsic academic motivation. To sum, this study contributed to previous research that confirmed that learners’ personality traits significantly influence learning motivation [83,84]. However, the findings in both sample groups are ambiguous, and the different patterns of links between personality traits and learning motivating factors in groups of participants and non-participants of e-learning based computer programming courses requires additional examination.

### 4.5. Self-Reported Cognitive Abilities Predict Learning Motivating Factors in Both Groups of Participants and Non-Participants of Computer Programming Courses

Furthermore, we assumed that self-reported cognitive abilities predict learning motivating factors in both groups of participants and non-participants of computer programming courses. Consequently, we conducted a multiple linear regression using learning motivating factors as the criterion and self-reported cognitive abilities as predictors in groups of respondents participating and not participating in e-learning based computer programming courses. The results partially confirmed this hypothesis. It demonstrated that in the group of computer programming e-learners, comprehension-knowledge was a significant predictor of individual attitude and expectation; fluid reasoning and comprehension-knowledge were significant predictors of challenging goals; comprehension-knowledge was a significant predictor of clear direction; visual processing was a significant predictor of social pressure and competition. On the other hand, in the group of non-participants of e-learning based computer programming courses, fluid reasoning was a significant predictor of individual attitude and expectation; fluid reasoning was a significant predictor of challenging goals; visual processing was a significant predictor of clear direction; visual processing was a significant predictor of reward and recognition. These findings are partially in line with some previous research [6,68,69,86,87,88,89,90]. However, the different patterns of links between self-reported cognitive abilities and learning motivating factors in groups of participants and non-participants of computer programming e-courses are unclear and need further investigation.

### 4.6. There Exist Associations between Learning Motivating Factors, Personality Traits, and Self-Reported Cognitive Abilities in Groups of Participants and Non-Participants of E-Learning Based Computer Programming Courses

Additionally, we assumed that there exist associations between learning motivating factors, personality traits, and self-reported cognitive abilities, but they differ between participants and non-participants of e-learning based computer programming courses. Thus, we conducted several SEM analyses to identify significant associations between the study variables in both sample groups. The findings revealed some statistically significant associations between the latent variables of personality traits, self-reported cognitive abilities, and learning motivating factors. This study also demonstrated that personality traits (namely, extraversion, conscientiousness, and reverted negative emotionality) statistically significantly predicts learning motivating factors (namely, individual attitude and expectation, and clear direction), and the impact of self-reported cognitive abilities in the model was negligible in both groups of participants and non-participants of e-learning based computer programming courses. These findings can be moderately supported by previous research which analyzed links between learning motivating factors, personality traits, and self-reported cognitive abilities [6,8,23,39,68,69,74,75,76,80,81,82,83,84,85,86,87,88,89,90]. This study at least modestly contributed to better understanding of the complexity of associations between intrinsic and extrinsic learning motivating factors, personality, and self-reported cognitive abilities. However, to comprehend the genuine links between e-learners learning motivation, personality traits, and cognitive abilities, it would be advantageous to apply neurocognitive methods, as self-reported measures, especially concerning cognitive abilities, might generate inaccurate results.

To sum, this study added to extensive research on learning motivation, personality, and cognitive abilities [4,5,8,23,39,68,69,74,76,78,89,90,107,108,109,110,111,112,113,114,115,116,117,118,119,120,121,122,123,124,125,126,127]. The findings revealed some distinct features of computer programming e-learners’ learning motivation, personality traits, and self-reported cognitive abilities. However, the results need further investigation, especially applying neurocognitive methods to assess genuine links between cognitive capabilities, personality traits, and learning.

### 4.7. Limitations and Future Directions

There are several limitations of this study that should be considered. First, as the objective cognitive capabilities were not measured, bias might occur due to the use of self-report measures only and omitting the objective indicators. Second, the findings should be regarded with caution as the data were collected online and based on self-observations. Third, the research samples were not representative but random, suggesting the necessity to analyze representative samples of e-learners; thus, generalizations should be made with caution. Fourth, this study was conducted in Lithuania, and the results might reflect the specifics of this area. Finally, the findings suggest a necessity for longitudinal or experimental research design, and we strongly suggest applying neurocognitive methods in future research, as self-report measures may generate inaccurate results.

### 4.8. Practical Implications

Computer programming competencies are among the fundamental skills that specialists are expected to acquire in the era of fast technology development [3], and hiring managers favor applicants who know programming languages and demonstrate computational literacy [2]. Educational organizations around the world encourage students to engage in programming learning [1], even though programming is acknowledged as one of the most difficult learning tasks [4], which relates to the high dropout rates and failures [5]. Research indicates that learning accomplishments might depend not only on numerous neurobiological factors, including genetic factors [9], cerebral metabolism [11,12], neurotrophic factors [14], neurotransmitters [13], hippocampal region [10], and the interactions between the distributed brain areas and neuronal networks [15,16,17,18,19,20]. Learning achievements might also depend on psychological factors, including learners’ characteristics [6] and learning motivation [4,7,8]. Thus, it was essential to identify links between computer programming e-learners’ personality traits, self-reported cognitive abilities, and learning motivating factors. The results revealed distinctive features of computer programming e-learners’ learning motivating factors, personality traits, and self-reported cognitive abilities. However, it also strongly suggested to apply neurocognitive methods in future research, as self-report measures may generate inaccurate results. Nonetheless, this research implies that education policymakers, neuroscience researchers, and educators, to promote learners’ developing computer programming skills, should target computer programming e-learners’ cognitive abilities, which are linked to personality traits and learning motivation.

## 5. Conclusions

This study aimed to identify computer programming e-learners’ personality traits, self-reported cognitive abilities, and learning motivating factors in comparison with a group of other e-learners. Computer programming e-learners demonstrated significantly lower extraversion scores than non-participants of e-learning based computer programming courses. Next, computer programming e-learners demonstrated significantly lower scores of motivating factors of individual attitude and expectation, reward and recognition, and punishment. In the group of respondents participating in computer programming courses, extraversion was a significant predictor of individual attitude and expectation; conscientiousness and extraversion were significant predictors of challenging goals; extraversion and agreeableness were significant predictors of clear direction; open-mindedness was a significant predictor of a diminished motivating factor of punishment; negative emotionality was a significant predictor of social pressure and competition. Next, in the group of computer programming e-learners, comprehension-knowledge was a significant predictor of individual attitude and expectation; fluid reasoning and comprehension-knowledge were significant predictors of challenging goals; comprehension-knowledge was a significant predictor of clear direction; visual processing was a significant predictor of social pressure and competition. The findings also revealed some statistically significant associations between the latent variables of personality traits, self-reported cognitive abilities, and learning motivating factors. This study demonstrated that personality traits (namely, extraversion, conscientiousness, and reverted negative emotionality) statistically significantly predict learning motivating factors (namely, individual attitude and expectation and clear direction). However, the impact of self-reported cognitive abilities in the model was negligible in both groups of participants and non-participants of e-learning based computer programming courses. However, as this study applied self-reported measures, the results should be taken with caution. We strongly suggest applying neurocognitive methods in future research to explore the genuine links between computer programming e-learners’ learning motivation, personality, and cognitive capabilities.

## Figures and Tables

**Table 1:** The learning motivating factors questionnaire: descriptive statistics and correlations between the subscales.

Learning Motivating Factors	Cronbach Alphas	M	SD	1	2	3	4	5
1. Individual attitude and expectation	0.815	4.71	0.86	-				
2. Challenging goals	0.870	4.33	1.08	0.407 ***	-			
3. Clear direction	0.697	4.97	0.78	0.648 ***	0.449 ***	-		
4. Reward and recognition	0.742	4.85	0.90	0.546 ***	0.118 *	0.504 ***	-	
5. Punishment	0.778	3.46	1.33	0.230 ***	0.158 **	0.257 ***	0.161 **	-
6. Social pressure and competition	0.825	3.46	1.21	0.295 ***	0.284 ***	0.199 ***	0.230 ***	0.422 ***

* *p* < 0.05; ** *p* < 0.01; *** *p* < 0.001.

**Table 2:** The BFI-2: descriptive statistics and correlations between the subscales.

The BFI-2 Subscales	Cronbach Alphas	M	SD	1	2	3	4
1. Extraversion	0.822	3.30	0.60	-			
2. Agreeableness	0.762	3.56	0.50	0.186 ***	-		
3. Conscientiousness	0.845	3.37	0.60	0.402 ***	0.331 ***	-	
4. Negative Emotionality	0.903	3.06	0.77	−0.379 ***	−0.272 ***	−0.326 ***	-
5. Open-Mindedness	0.683	3.65	0.46	0.375 ***	0.157 ***	0.196 ***	−0.037

*** *p* < 0.001.

**Table 3:** The SRMCA: descriptive statistics and correlations between the subscales.

The SRMCA Subscales	Cronbach Alphas	M	SD	1	2
1. Fluid reasoning (Gf)	0.809	5.13	0.95	-	
2. Comprehension-knowledge (Gc)	0.776	5.12	1.00	0.685 ***	-
3. Visual processing (Gv)	0.783	5.46	0.85	0.630 ***	0.511 ***

*** *p* < 0.001.

**Table 4:** Differences in personality traits between the groups of participants and non-participants of e-learning based computer programming courses.

							95% CI for Cohen’s d	
Personality Traits	*t*	df	*p*	Mean Difference	SE Difference	Cohen’s d	Lower	Upper
Extraversion	−2.433	478	0.015	−0.135	0.055	−0.227	−0.411	−0.044
Agreeableness	0.715	478	0.475	0.034	0.047	0.067	−0.117	0.250
Conscientiousness	1.132	478	0.258	0.064	0.057	0.106	−0.078	0.289
Negative emotionality	−0.627	478	0.531	−0.045	0.072	−0.059	−0.242	0.125
Open-mindedness	0.118	478	0.906	0.005	0.043	0.011	−0.172	0.194

**Table 5:** Differences in self-reported cognitive abilities between the groups of participants and non-participants of e-learning based computer programming courses.

							95% CI for Cohen’s d	
Self-Reported Cognitive Abilities	t	df	*p*	Mean Difference	SE Difference	Cohen’s d	Lower	Upper
Fluid reasoning	−0.403	453	0.687	−0.037	0.091	−0.038	−0.226	0.149
Comprehension knowledge	−0.808	453	0.420	−0.077	0.095	−0.077	−0.264	0.110
Visual processing	0.962	453	0.336	0.078	0.081	0.092	−0.095	0.279

**Table 6:** Differences in learning motivating factors between the groups of participants and non-participants of e-learning based computer programming courses.

							95% CI for Cohen’s d	
Learning Motivating Factors	*t*	df	*p*	Mean Difference	SE Difference	Cohen’s d	Lower	Upper
Individual attitude and expectation	−2.875	403	0.004	−0.245	0.085	−0.287	−0.484	−0.090
Challenging goals	0.569	403	0.570	0.062	0.108	0.057	−0.139	0.253
Clear direction	−1.929	403	0.054	−0.150	0.078	−0.193	−0.389	0.004
Reward and recognition	−3.092	403	0.002	−0.276	0.089	−0.309	−0.505	−0.112
Punishment	−3.095	403	0.002	−0.407	0.131	−0.309	−0.506	−0.112
Social pressure and competition	−1.647	403	0.100	−0.198	0.120	−0.164	−0.360	0.032

**Table 7:** Multiple linear regression models in the group of computer programming e-learners, the dependent variables are learning motivating factors, and the predictors are personality traits.

Dependent Variables	Predictors/ Models	Unstandardized Coefficients	Standardized Coefficients		*t*	Sig.	R	R^2^	F	Sig.
		B	Std. Error	Beta						
Individual attitude and expectation	1 (Constant)	3.039	0.341		8.917	0.000	0.326	0.106	21.373	0.000
	Extraversion	0.482	0.104	0.326	4.623	0.000				
Challenging goals	1 (Constant)	1.610	0.394		4.086	0.000	0.469	0.220	50.661	0.000
	Conscientiousness	0.810	0.114	0.469	7.118	0.000				
	2 (Constant)	1.026	0.435		2.356	0.020	0.505	0.255	30.600	0.004
	Conscientiousness,	0.645	0.125	0.373	5.156	0.000				
	Extraversion	0.357	0.123	0.210	2.906	0.004				
Clear direction	1 (Constant)	3.527	0.295		11.968	0.000	0.331	0.110	22.173	0.000
	Extraversion	0.424	0.090	0.331	4.709	0.000				
	2 (Constant)	2.452	0.459		5.337	0.000	0.390	0.152	16.101	0.003
	Extraversion,	0.391	0.089	0.305	4.398	0.000				
	Agreeableness	0.330	0.110	0.209	3.007	0.003				
Punishment	1 (Constant)	4.838	0.767		6.306	0.000	0.155	0.024	4.440	0.037
	Open-mindedness	−0.439	0.209	−0.155	−2.107	0.037				
Social pressure and competition	1 (Constant)	4.048	0.326		12.419	0.000	0.161	0.026	4.810	0.030
	Negative emotionality	−0.227	0.104	−0.161	−2.193	0.030				

**Table 8:** Multiple linear regression models in the group of non-participants of computer programming courses, the dependent variables are learning motivating factors, and the predictors are personality traits.

Dependent Variables	Predictors/ Models	Unstandardized coefficients	Standardized Coefficients		*t*	Sig.	R	R^2^	F	Sig.
		B	Std. Error	Beta						
Individual attitude and expectation	1 (Constant)	3.898	0.300		13.007	0.000	0.207	0.043	9.866	0.002
	Conscientiousness	0.276	0.088	0.207	3.141	0.002				
Challenging goals	1 (Constant)	2.687	0.411		6.537	0.000	0.261	0.068	160.021	0.000
	Conscientiousness	0.483	0.121	0.261	4.003	0.000				
	2 (Constant)	3.837	0.589		6.515	0.000	0.313	0.098	11.861	0.000
	Conscientiousness,	0.400	0.123	0.216	3.255	0.001				
	Negative emotionality	−0.283	0.105	−0.178	−2.692	0.008				
Clear direction	1 (Constant)	3.694	0.312		11.848	0.000	0.283	0.080	19.116	0.000
	Extraversion	0.395	0.090	0.283	4.372	0.000				
	2 (Constant)	3.286	0.351		9.361	0.000	0.323	0.104	12.714	0.000
	Extraversion,	0.300	0.097	0.215	3.082	0.002				
	Conscientiousness	0.217	0.090	0.169	2.426	0.016				
Reward and recognition	1 (Constant)	4.019	0.323		12.457	0.000	0.198	0.039	9.011	0.000
	Conscientiousness	0.284	0.095	0.198	3.002	0.003				

**Table 9:** Multiple linear regression models in group of participants of computer programming courses, the dependent variables are learning motivating factors, and the predictors are self-reported cognitive abilities.

Dependent Variables	Predictors/ Models	Unstandardized Coefficients	Standardized Coefficients		*t*	Sig.	R	R^2^	F	Sig.
		B	Std. Error	Beta						
Individual attitude and expectation	1 (Constant)	3.577	0.359		9.963	0.000	0.206	0.043	8.060	0.005
	Comprehension-knowledge	0.197	0.070	0.206	2.839	0.005				
Challenging goals	1 (Constant)	1.941	0.384		5.061	0.000	0.431	0.186	41.361	0.000
	Fluid reasoning	0.477	0.074	0.431	6.431	0.000				
	2 (Constant)	1.428	0.412		3.467	0.001	0.475	0.225	26.194	0.000
	Fluid reasoning,	0.299	0.093	0.270	3.204	0.002				
	Comprehension-knowledge	0.279	0.092	0.256	3.027	0.003				
Clear direction	1 (Constant)	3.673	0.302		12.144	0.000	0.291	0.085	16.734	0.000
	Comprehension-knowledge	0.240	0.059	0.291	4.091	0.000				
Social pressure and competition	1 (Constant)	1.951	0.569		3.428	0.001	0.182	0.033	6.212	0.014
	Visual processing	0.256	0.103	0.182	2.492	0.014				

**Table 10:** Multiple linear regression models in the group of non-participants of computer programming courses, the dependent variables are learning motivating factors, and the predictors are self-reported cognitive abilities.

Dependent Variable	Predictors/ Models	Unstandardized Coefficients	Standardized Coefficients		*t*	Sig.	R	R^2^	F	Sig.
		B	Std. Error	Beta						
Individual attitude and expectation	(Constant)	3.961	0.297		13.347	0.000	0.195	0.038	8.739	0.003
	Fluid reasoning	0.168	0.057	0.195	2.956	0.003				
Challenging goals	(Constant)	2.899	0.409		7.081	0.000	0.229	0.053	12.212	0.001
	Fluid reasoning	0.273	0.078	0.229	3.495	0.001				
Clear direction	(Constant)	4.243	0.343		12.356	0.000	0.156	0.024	5.491	0.020
	Visual processing	0.146	0.062	0.156	2.343	0.020				
Reward and recognition	(Constant)	3.750	0.379		9.901	0.000	0.215	0.046	10.644	0.001
	Visual processing	0.225	0.069	0.215	3.263	0.001				

**Table 11:** Scalar estimates of the model on associations between learning motivating factors, self-reported cognitive abilities, and personality traits in both groups of participants and non-participants of computer programming e-learning based courses.

Regression			B	S.E.	Z	*p*	LL	UL	β	Group *
Personality traits	→	Learning motivating factors	0.660	0.235	2.810	0.005	0.200	1.121	0.460	1
Self-reported cognitive abilities	→	Learning motivating factors	−0.015	0.101	−0.152	0.879	−0.213	0.182	−0.021	1
Personality traits	→	Learning motivating factors	0.599	0.189	3.164	0.002	0.228	0.971	0.427	2
Self-reported cognitive abilities	→	Learning motivating factors	0.033	0.061	0.549	0.583	−0.086	0.153	0.048	2
**Measurement model**										
Self-reported cognitive abilities	→	Fluid reasoning	1.000	0.000			1.000	1.000	0.899	1
Self-reported cognitive abilities	→	Visual processing	0.595	0.077	7.685	<0.001	0.443	0.747	0.600	1
Self-reported cognitive abilities	→	Comprehension-knowledge	0.797	0.092	8.661	<0.001	0.617	0.978	0.713	1
Personality traits	→	Extraversion	1.000	0.000			1.000	1.000	0.705	1
Personality traits	→	Negative emotionality	−1.204	0.188	−6.405	<0.001	−1.573	−0.836	−0.621	1
Personality traits	→	Conscientiousness	0.920	0.129	7.105	<0.001	0.666	1.174	0.660	1
Learning motivating factors	→	Individual attitude and expectation	1.000	0.000			1.000	1.000	0.683	1
Learning motivating factors	→	Clear direction	1.247	0.257	4.845	<0.001	0.742	1.751	0.983	1
Self-reported cognitive abilities	→	Fluid reasoning	1.000	0.000			1.000	1.000	0.883	2
Self-reported cognitive abilities	→	Visual processing	0.754	0.062	12.128	<0.001	0.632	0.875	0.756	2
Self-reported cognitive abilities	→	Comprehension-knowledge	1.007	0.071	14.201	<0.001	0.868	1.145	0.813	2
Personality traits	→	Extraversion	1.000	0.000			1.000	1.000	0.744	2
Personality traits	→	Negative emotionality	−0.789	0.150	−5.266	<0.001	−1.082	−0.495	−0.462	2
Personality traits	→	Conscientiousness	0.787	0.155	5.077	<0.001	0.483	1.091	0.539	2
Learning motivating factors	→	Individual attitude and expectation	1.000	0.000			1.000	1.000	0.721	2
Learning motivating factors	→	Clear direction	1.154	0.264	4.371	<0.001	0.637	1.672	0.864	2

* Group 1: participants of e-learning based computer programming courses; Group 2: non-participants of e-learning based computer programming courses.

## Data Availability

Data are available upon request from the corresponding author.
